# Trends in Liver Cancer Incidence and Survival in Italy by Histologic Type, 2003–2017

**DOI:** 10.3390/cancers14246162

**Published:** 2022-12-14

**Authors:** Silvia Mancini, Lauro Bucchi, Federica Zamagni, Stefano Guzzinati, Luigino Dal Maso, Massimo Rugge, Lucia Bisceglia, Diego Serraino, Claudia Casella, Adele Caldarella, Fabio Falcini, Antonino Musolino, Giuliano Carrozzi, Roberto Vito Rizzello, Lucia Mangone, Guido Mazzoleni, Pietro Seghini, Stefano Ferretti, the Italian Cancer Registries’ Collaborative Group

**Affiliations:** 1 Emilia-Romagna Cancer Registry, Romagna Unit, Romagna Cancer Institute, IRCCS Istituto Romagnolo per lo Studio dei Tumori (IRST) Dino Amadori, 47014 Meldola, Italy; 2 Veneto Tumour Registry, Azienda Zero, 35132 Padova, Italy; 3 Cancer Epidemiology Unit, Centro di Riferimento Oncologico di Aviano (CRO) IRCCS, 33081 Aviano, Italy; 4 Department of Medicine (DIMED) - Università degli Studi di Padova, 35122 Padova, Italy; 5 Cancer Registry of Puglia, 70126 Bari, Italy; 6 Liguria Cancer Registry, IRCCS Ospedale Policlinico San Martino, 16132 Genoa, Italy; 7 Tuscany Cancer Registry, Clinical Epidemiology Unit, Institute for Cancer Research, Prevention and Clinical Network (ISPRO), 50139 Florence, Italy; 8 Local Health Authority, 47121 Forlì, Italy; 9 Emilia-Romagna Cancer Registry, Parma Unit, Medical Oncology Unit, University Hospital of Parma, 43126 Parma, Italy; 10 Emilia-Romagna Cancer Registry, Modena Unit, Public Health Department, Local Health Authority, 41121 Modena, Italy; 11 Trento Province Cancer Registry, Unit of Clinical Epidemiology, 38123 Trento, Italy; 12 Emilia-Romagna Cancer Registry, Reggio Emilia Unit, Epidemiology Unit, Azienda Unità Sanitaria Locale–IRCCS di Reggio Emilia, 42122 Reggio Emilia, Italy; 13 South Tyrol Cancer Registry, 39100 Bolzano, Italy; 14 Emilia-Romagna Cancer Registry, Piacenza Unit, Public Health Department, AUSL Piacenza, 29121 Piacenza, Italy; 15 Emilia-Romagna Cancer Registry, Ferrara Unit, Local Health Authority, Ferrara; and University of Ferrara, 44121 Ferrara, Italy

**Keywords:** liver cancer, hepatocellular carcinoma, intrahepatic cholangiocarcinoma, incidence, survival, age–period–cohort modelling, trend

## Abstract

**Simple Summary:**

We evaluated recent trends in liver cancer incidence and survival in Italy (2003–2017) based on data from 13 cancer registries covering 21% of the national population. The incidence of total liver cancer and hepatocellular carcinoma (HCC) decreased significantly for both sexes. Intrahepatic cholangiocarcinoma (ICC) followed an opposite trend. The risk of HCC saw a peak for people born around 1930 and another, more moderate peak for those born in the late 1950s. Men and women exhibited comparable improvements in both early and mid-term (conditional) net survival from HCC. The uptrend in survival from ICC was less pronounced. The opposite incidence trends of HCC and ICC confirm a pattern previously observed in other populations. The generalised, albeit slow, improvement in survival from HCC indicates a trend towards an earlier detection coupled with improvements in treatments.

**Abstract:**

(1) Background: Liver cancer in Italy is characterised by one of the highest incidence rates worldwide outside of Asia coupled with comparatively favourable survival figures. The objective of this study was to evaluate the most recent epidemiologic trends of the disease. (2) Methods: Thirteen cancer registries covering a population of about 12,740,000 (21% of the national population) made available the records of 35,574 cases registered between 2003 and 2017. Trends in age-standardised (Europe 2013) incidence rates were analysed using the results of age–drift models. Trends in survival were analysed using 1-year, 2-year, 5-year and 10-year net survival (NS) and 5|1-year and 5|2-year conditional NS. (3) Results: Over the study period, the average annual incidence rates per 100,000 persons were 29.4 (men) and 9.4 (women) for total liver cancer; 14.6 and 3.5 for hepatocellular carcinoma (HCC); 1.8 and 1.1 for intrahepatic cholangiocarcinoma (ICC); and 13.0 and 4.8 for the ‘other liver cancer types’ group. The incidence of total liver cancer and HCC decreased significantly for both sexes. For total liver cancer, the estimated average annual percent change was −1.6% among men and −2.1% among women. For HCC, the change was −1.3% among men and −2.7% among women. ICC followed an opposite trend. For men, the risk of HCC had two peaks, one in the birth cohorts of 1928 and 1933 and another, more moderate peak in the cohort of 1958. Men and women exhibited comparable improvements in both early and mid-term conditional NS from HCC. In 2013–2017, 5-year NS was 28.9% (95% CI: 27.3%; 30.6%) for men and 30.1% (95% CI: 26.9%; 33.5%) for women. The uptrend in survival from ICC was less pronounced and was weakly significant, with a 5-year NS in 2013-2017 of 13.9% (95% CI: 10.8%; 17.3%) for men and 17.4% (95% CI: 13.5%; 21.7%) for women. (4) Conclusions: The opposite incidence trends of HCC and ICC confirm a pattern observed in other populations. The generalised, albeit slow, improvement in survival from HCC indicates a trend towards earlier detection coupled with improvements in treatments.

## 1. Introduction

Liver cancer comprises two main histologic types, hepatocellular carcinoma (HCC) and intrahepatic cholangiocarcinoma (ICC), and other rare primary liver malignancies. Many modifiable and non-modifiable risk factors for HCC, ICC or both have been reported, although the molecular pathways by which they cause liver cancer are not fully understood [1]. The two diseases have a substantially different aetiology. The major risk factors for HCC, which accounts for an average of approximately 80% of incidence, are hepatitis B and C virus (HBV and HCV) infection—through unsafe injections and other healthcare-related practices, or intravenous drug use or sexual contact—and, less frequently, the ingestion of aflatoxins, alcohol abuse, diabetes, obesity and non-alcoholic fatty liver disease. These factors are predisposing conditions for cirrhosis, the precursor of most HCC cases [2,3,4].

With respect to the less common ICC, the majority of cases occur in patients with no known or suspected risk factors [5]. The only established causes are food-borne parasites, which are found in endemic areas, particularly in eastern Asia and the Russian Federation [4]. In non-endemic areas, only a minority of risk factors for ICC overlap with those for HCC [6]. The main ones are biliary cysts and stones, cirrhosis, sclerosing cholangitis [7], HBV and HCV [8]. Recent observations have suggested an important aetiologic role for overweight, obesity, metabolic syndrome and non-alcoholic fatty liver disease [9,10,11].

As a consequence of different time trends in the prevalence of risk factors, HCC and ICC also differ with respect to time trends in incidence. For decades, many studies have reported a substantial increase in the rates of HCC all over the world, which has led to an increase in overall liver cancer rates. The steepest trend slopes have been observed in eastern and south-eastern Asia, northern Africa and Oceania [3,4,12], but incidence has also increased in North America and Europe [3]. According to more recent reports, the rates have peaked and are now plateauing or decreasing among younger and middle-aged adults, at least in the male population. For example, this is the case for several eastern and south-eastern Asian countries [13,14], the UK [5] and the United States [2,15,16,17], where the trend has involved high-risk immigrants [18]. Given the complex aetiology of the disease, this trend may have at least partially different explanations in different geographic areas. The main factors involved may vary from reduced aflatoxin exposure to improvements in HBV vaccination programmes and in treatment for HBV and HCV infections.

ICC has followed an incidence trend in the opposite direction. In the United States in particular, the rates have shown a remarkable increase during the past decade [6,19]. The geographic epidemiology of ICC, too, is different, because the disease is more frequent in eastern and south-eastern Asia, but also in Europe and central America [4]. Further differences between HCC and ICC can be observed for demographic, socioeconomic [20] and clinical characteristics, including tumour stage at diagnosis and survival [9,11]. Population-based survival from ICC, in fact, has been the subject of a few investigations. In Europe, survival from liver cancer as a whole began to increase moderately in the last decade of the 1990s [21]. The CONCORD-3 global study confirmed that, for most countries, net survival (NS) from liver cancer has changed little between 1995 and 2014, although with some differences [22]. In any case, these improvements have been confined to, or have been mostly accounted for by, HCC. In the United States, a study based on data from the Surveillance, Epidemiology, and End Results database found no significant change in survival from ICC between 1975 and 2016 [19], with a substantial improvement being reported only in patients undergoing surgical treatment [23]. A moderately favourable trend has been observed in some European countries during the past two decades [5,24,25].

The descriptive epidemiology of liver cancer in Italy has remarkable peculiarities. Despite a levelling-off observed in the last years of the twentieth century [26], the incidence remains very high and is still among the highest worldwide outside of Asia [3]. Among the 27 countries participating in the European Cancer Information System [27], Italy ranks third (men) and second (women) for the total age-standardised liver cancer incidence rate per 100,000 persons (men: 24.9 in a range of 11.2–28.5; women: 9.4 in a range of 4.6–11.3). The ranking is similar for the percent proportion of liver cancer cases out of total incident cancers (all sites but non-melanoma skin) (men: second with 3.9 in a range of 1.4–4.6; women: third with 2.1 in a range of 1.0–2.8). Italy is also characterised by comparatively favourable survival figures. Between 1995 and 2014, the 5-year NS probability has increased and, for patients diagnosed between 2010 and 2014, has reached above 20%, which can be considered a high level on a European and a global scale [22]. Finally, the proportion of liver cancers due to HCV infection is as high as 50–60% versus a global average of 19% [28]. These considerations provided the rationale for the present study, a population-based study aimed at estimating the trends in liver cancer incidence and survival in Italy, overall and by major histologic type, using the most recent data available (2003–2017).

## 2. Materials and Methods

### 2.1. Rationale and Design

We assumed that the differences reported to date in risk factors, incidence, survival and time trends [1,3,4,5,6,7,8,9,10,13,15,16,17,18,19] are of sufficient significance and importance to establish that HCC and ICC are substantially different aetiologically, biologically and clinically. With respect to total liver cancer (all types combined), we estimated only the time trends in the annual incidence rate as information of interest from a general public health point of view.

### 2.2. Data

Thirteen cancer registries contributed to the present study, covering the administrative regions of Friuli Venezia Giulia, Veneto (43% coverage), Romagna (100%) and Puglia (69%), and the provinces of Trento, Bolzano (South Tyrol Cancer Registry), Ferrara, Modena, Reggio Emilia, Parma, Piacenza, Firenze, Prato, and Genova. Table 1 shows their population and the case series contributed to the study (years of registration, number, percentage of microscopic verification and of death-certificate-only, or DCO, cases). At the end of the local period of registration, the size of the populations varied from approximately 290,000 to 2.8 million for a total of about 12,740,000 or 21% of the Italian national population. Supplementary Figure S1 shows their geographic distribution.

At the time this study was designed, the participating registries had collected incidence data for a number of years of registration varying from 12 to 40. For the study, we selected the same 15-year time period of cancer registration, 2003–2017, that is covered by *Cancer Incidence in Five Continents* Volumes X (2003–2007) [29] and XI (2008-2012) [30] and by the forthcoming Volume XII (2013-2017) [31]. The registries contributed data for the entire study period with the exception of those situated in the Region of Puglia and in the province of Piacenza, which were not yet in operation during the initial three years, and the registries of Firenze-Prato and Genova, which did not provide data for the year 2017 (Table 1). In addition, the data collection by the registry of Firenze-Prato was not done in 2012 because the system was rearranged in order to expand the registration area to the whole surrounding administrative region (data for 2012 are being retrospectively retrieved but are still incomplete).

The registries made available the anonymous records of 35,574 incident cases of malignant neoplasms of the liver (*International Classification of Diseases for Oncology*, 3rd edition, 1st revision, topography code C22) [32] with morphologies grouped as HCC (codes 8170–8175, 8970); ICC (codes 8013, 8020, 8041, 8154, 8160–8162, 8180, 8240, 8246, 8249, 8470) [33]; and “other carcinomas and unspecified neoplasia types” (hereby referred to as ‘other liver cancer types’) (codes 8000–8004, 8010, 8011, 8021, 8032–8034, 8140, 8141, 8190, 8230, 8260, 8310). No other types of cancer were considered. The proportion of cases verified by histology was 40%. The ‘other liver cancer types’ group (*n* = 16,668) was mostly comprised of cases without histological verification (morphology code 8000) (*n* = 15,001 or 90.0%). The proportion registered based on death certificate only was 2.0%. (Table 1).

### 2.3. Statistical Methods

#### 2.3.1. Incidence

The annual and 5-year incidence rates per 100,000 persons were age-standardised to the 2013 European standard population using the direct method. Ninety-five percent confidence intervals (CI) were computed according to the Poisson distribution.

We performed an age–period–cohort analysis [34,35,36] of the incidence trends. We first used age–period–cohort modelling to explore the time trend in the age-standardised incidence rates of total liver cancer, HCC, ICC and ‘other liver cancer types’. To assess the magnitude and direction of the trend, we used the results of the age–drift model. The net drift parameter is a one-degree-of-freedom linear term for time representing the estimated annual percent change (EAPC) in the rates over time. The drift is common to calendar period and birth cohort. The EAPC is linear on a log scale and can be used for comparisons irrespective of the magnitude of the rates at baseline [37].

Subsequently, we restricted the age–period–cohort analysis to the population aged 45–84 years. People aged <45 years were excluded due to the extreme rarity of HCC, ICC and ‘other liver cancer types’ in these age groups, while those aged >84 years were excluded because of the instability of rates. This left a population of 2,955,716 men and 3,273,386 women available for analysis, with 14,907 HCC cases (11,527 men and 3380 women), 2467 ICC cases (1431 men and 1036 women) and 13,670 ‘other liver cancer types’ cases (9445 men and 4225 women).

The data for HCC, ICC and ‘other liver cancer types’ were tabulated into eight 5-year age groups, three 5-year time periods of diagnosis (namely: 2003–2007, 2008–2012, 2013–2017) and ten overlapping 10-year birth cohorts, which were identified by their mid-year of birth. The age-specific rates were plotted against the period and the cohort.

For HCC and ICC, five Poisson regression models were fitted according to the model-building approach [35]. The model goodness-of-fit was assessed using residual deviance statistics. The models were compared using conditional likelihood ratio tests between hierarchically nested models and the Akaike information criterion [38]. Further details of model fitting are provided in Section 3.

The best-fitting models included age and cohort (HCC, men) and age and drift (HCC, women; ICC, both sexes). The cohort of 1928, the one at highest risk, was used as a reference. The cohort effect was interpreted as an incidence rate ratio (IRR). The age effect was interpretable as an age-specific incidence rate for the reference cohort. The age and cohort effect estimates were modelled by means of restricted cubic spline functions. We used a spline function with five internal knots for the age and cohort variables. Pointwise CIs were computed. Unlike the complete age–period–cohort model, the age–cohort and the age–drift models do not suffer from the problem of non-identifiability of parameters.

#### 2.3.2. Survival

Patients were followed up until 31 December 2018. DCO cases (*n* = 714) and patients aged <15 years (*n* = 12) [22] were excluded from the survival analysis. This left 16,154 HCC cases (12,305 men and 3849 women), 2679 ICC cases (1531 men and 1148 women) and 16,015 ‘other liver cancer types’ cases (10,498 men and 5517 women).

We explored time trends in survival using multiple prognostic indicators, namely: 1-year, 2-year, 5-year and 10-year NS and two measures of 5-year conditional NS (CNS). NS is defined as the probability of surviving cancer in the absence of other causes of death, that is, the survival that would be observed if liver cancer was the only possible cause of death. By implication, NS is not influenced by cross-sectional differences and temporal changes in mortality from other causes. This enables performing unbiased survival comparisons between subpopulations and across time. We used two indicators of CNS, namely: the probability of surviving an additional 4 years on the condition that the patient has survived 1 year, and the probability of surviving an additional 3 years on the condition that the patient has survived 2 years, hereby referred to as 5|1-year CNS and 5|2-year CNS, respectively.

The above measures of NS inform about the effect of distinct clinical prognostic factors, allowing to disentangle early from later survival improvements (if any). One- and 2-year survival rates are adversely affected by the prevalence of late-stage, rapidly fatal cancers, which indicates diagnostic delays or problems with the referral pathway. Improvements in 1-year and 2-year NS are improvements in early survival and reflect a downstaging of the disease [39]. CNS indicators, conversely, must be considered mid-term outcome measures. They are impacted by more delayed fatalities caused by the growth of occult metastases at diagnosis. Consequently, they are more sensitive to improvements in adjuvant and supportive treatments.

One-year, 2-year, 5-year and 10-year NS rates were calculated using the Pohar-Perme estimator [40]. The estimates were age-standardised using the International Cancer Survival Standard (ICSS)-1 weights [41]. The 5|1-year and 5|2-year CNS with the 95% CI were obtained from the NS at 1 + 4 years and 2 + 3 years after diagnosis, with the time at risk being computed from one and two years after diagnosis [42]. To adjust for the background mortality, administrative region-specific lifetables, stratified by year, patient age and sex, from the Italian National Statistics Institute were used.

Follow up ended on 31 December 2018, and, thus, all patients diagnosed during 2003–2007 contributed to the estimate of survival up to 10 years after diagnosis. We used the cohort approach to estimate NS in this time period [43]. Instead, a hybrid approach was used to estimate NS for the periods 2008-2012 and 2013-2017, because not all patients were followed for two, five or 10 years. This method is a combination of the period and cohort methods [44].

The statistical significance of all trends in all survival indicators was assessed with Poisson regression models including the period of diagnosis as a continuous regressor. Specifically, the statistical significance was set at the 95% confidence level (*p*-value < 0.05) and was assessed with the Wald test for trend. A borderline statistical significance was defined as a *p*-value between 0.05 and 0.10.

For both incidence and survival estimates, the STATA package version 15.1 (Stata Corporation, College Station, TX, USA) was used.

## 3. Results

### 3.1. Incidence

Table 2 shows the distribution of the 35,574 study cases by sex, disease type and period of diagnosis. Overall (column at left), the cases comprised 24,692 (69.4%) men and 10,882 (30.6%) women. The men-to-women IRR was 4.21 (95% CI: 4.06; 4.37) for HCC and 1.65 (95% CI: 1.53; 1.78) for ICC. The proportion of HCC out of total cases of known type was 12,343/13,874 (89.0%) among men and 3881/5032 (77.1%) among women. In Table 2, the average annual age-standardised incidence rates by time period are also shown, suggesting a decreasing trend for total liver cancer, HCC and ‘other liver cancer types’ and an increase for ICC.

**Figure 1:**
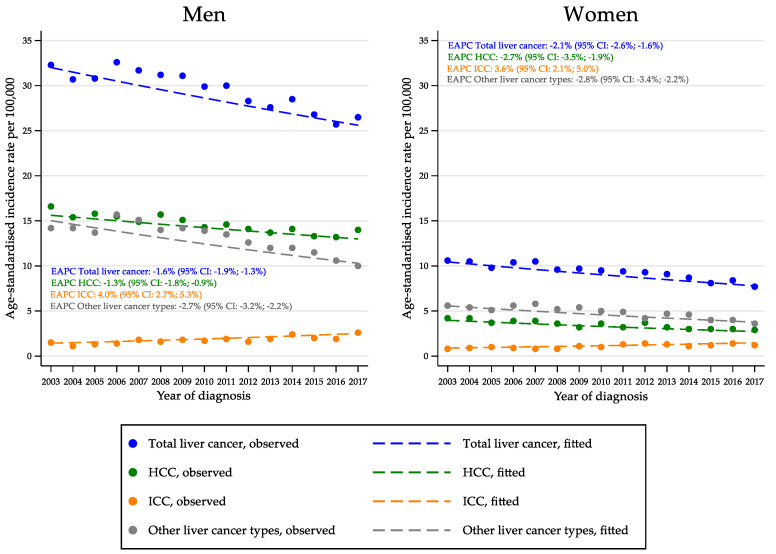
Annual age-standardised incidence rates of total liver cancer, hepatocellular carcinoma, intrahepatic cholangiocarcinoma, ‘other liver cancer types’ and estimated annual percent change, by sex (Italy, 2003–2017). CI, confidence interval; EAPC, estimated annual percent change; HCC, hepatocellular carcinoma; ICC, intrahepatic cholangiocarcinoma. A fitted line was added to the observed values (points).

To evaluate the time trend in age-standardised incidence rates between 2003 and 2017, the results of the age–drift model were used (Figure 1). Among men, a significant downward trend was found for total liver cancer (EAPC: −1.6%; 95% CI: −1.9%; −1.3%), HCC (EAPC: −1.3%; 95% CI: −1.8%; −0.9%) and ‘other liver cancer types’ (EAPC: −2.7%; 95% CI: −3.2%; −2.2%). ICC displayed a significant opposite trend (EAPC: 4.0%; 95% CI: 2.7%; 5.3%). Among women the results were similar, with an EAPC of −2.1% (95% CI: −2.6%; −1.6%) for total liver cancer; −2.7% (95% CI: −3.5%; −1.9%) for HCC; −2.8% (95% CI: −3.4%; −2.2%) for ‘other liver cancer types’; and 3.6% (95% CI: 2.1%; 5.0%) for ICC.

**Figure 2:**
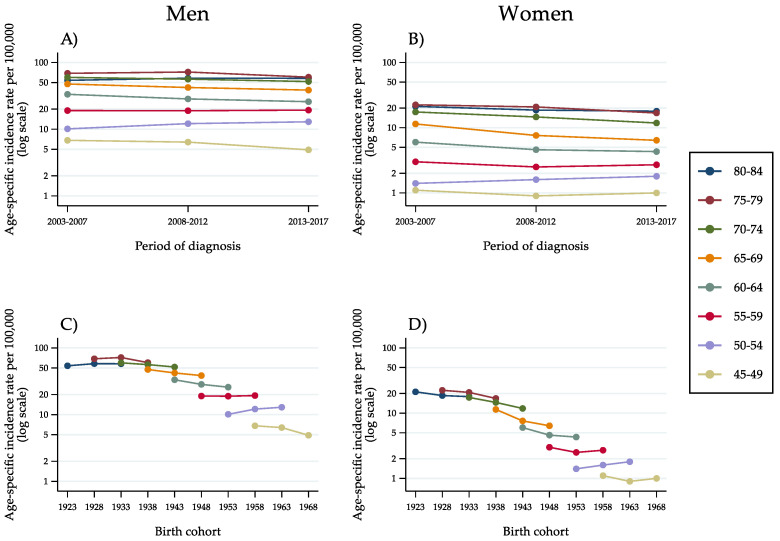
Age-specific incidence rates of hepatocellular carcinoma in the population aged 45–84 years, by 5-year age group, 5-year period of diagnosis (**A**,**B**) and 10-year birth cohort (**C**,**D**) as identified by the mid-year of birth (Italy, 2003–2017).

All subsequent analyses were restricted to the age range 45–84 years. Figure 2 shows the age-specific incidence rate of HCC by 5-year age group, 5-year period of diagnosis (Figure 2A,B) and 10-year birth cohort (Figure 2C,D). Among men, the curves were roughly parallel and peaked at approximately 70 per 100,000 men at age 75–79 years (Figure 2A,C). Incidence rates by time period decreased consistently in the age groups above 60 years. In the population aged 55–59 years, a stabilisation occurred, followed by a moderate increase in the age group 50–54 years. This is suggestive of an interaction between period and age, that is, a non-linear cohort effect. For women, the rates were considerably lower and less stable. The pattern of trends, however, was comparable to that of men.

Incidence curves by age group for successive cohorts, on a semi-logarithmic scale, showed a decline involving virtually all men born between 1923 and 1948 (Figure 2C). The risk stabilised for the cohort born in 1953 and rose for the cohort of 1958. Subsequently, incidence began decreasing again among men born in 1963. Even though at a considerably lower level of risk, women showed a comparable pattern of findings. A decreasing incidence trend involved all cohorts up to, and including, the cohort of 1953. An opposite trend was observed among women born in 1958. Like among men, the change was transient. For the cohort born in 1963, a stabilisation occurred.

**Figure 3:**
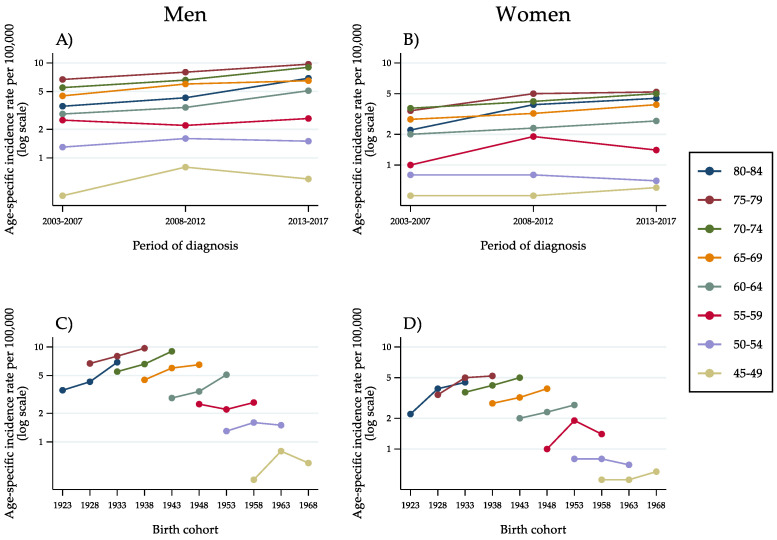
Age-specific incidence rates of intrahepatic cholangiocarcinoma in the population aged 45–84 years, by 5-year age group, 5-year period of diagnosis (**A**,**B**) and 10-year birth cohort (**C**,**D**) as identified by the mid-year of birth (Italy, 2003–2017).

With respect to ICC (Figure 3A–D), the rates were lower and suffered from marked instability, especially in the female population and in the age groups below 55 years (Figure 3B,D). In the older age groups, for whom the rates were higher, an upward trend was evident, with an almost linear increase in age-specific incidence over time.

**Figure 4:**
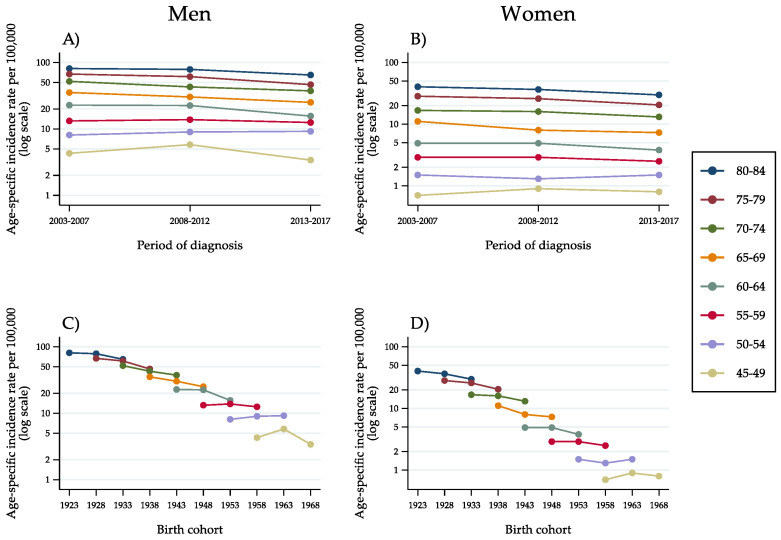
Age-specific incidence rates of ‘other liver cancer types’ in the population aged 45–84 years, by 5-year age group, 5-year period of diagnosis (**A**,**B**) and 10-year birth cohort (**C**,**D**) as identified by the mid-year of birth (Italy, 2003–2017).

Regarding ‘other liver cancer types’ (Figure 4A–D), the pattern of incidence trends was similar to that observed for HCC, with diminishing rates up to, and including, the age group of 60–64 years. For lower age groups, that is, 55–59 years and 50–54 years, a stabilisation and a slight increase occurred.

As far as the age–period–cohort modelling analysis is concerned, the age–cohort model provided the best fit to the HCC incidence data in the male population (Table 3).

**Figure 5:**
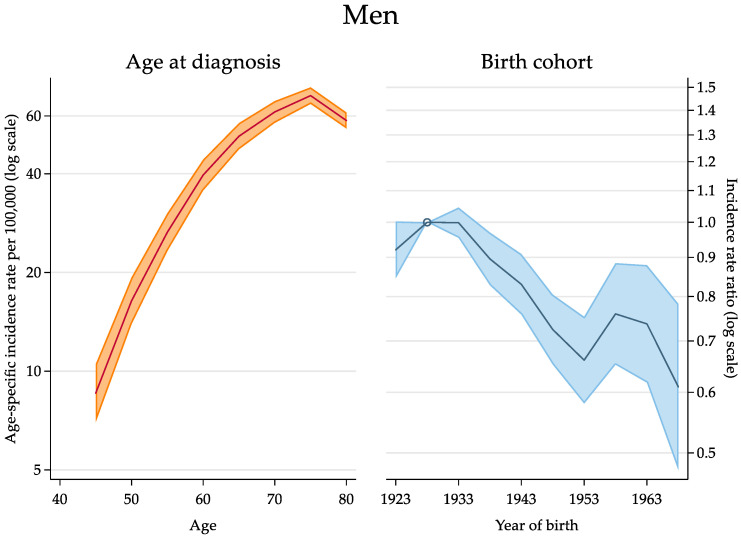
Age-specific incidence rate of hepatocellular carcinoma per 100,000 persons in the male population by age at diagnosis (panel at left) and incidence rate ratio by birth cohort (panel at right), as identified by the mid-year of birth (Italy, 2003–2017). The cohort of 1928, at highest risk, was the reference cohort. Incidence rate and rate ratio estimates (thick lines) and 95% confidence intervals (area around) are shown on a log scale.

The relative contribution of the estimated effects of age and cohort is depicted in Figure 5. Compared with the cohort of 1928, for which the risk peaked, the estimated IRR was virtually equal in the cohort of 1933. A considerable decrease occurred until the cohort born in 1953, with an IRR of 0.66 (95% CI: 0.58; 0.75). A second peak at 0.76 was reached in the cohort of 1958, with a very similar figure in the subsequent one (1963). Then, a new decrease took place. The estimated values of IRRs by birth cohort are detailed in Table A1 (Appendix A). With respect to women, the age–drift model provided the best fit to the data thanks to a lower Akaike information criterion score. There were no remarkable and significant period and cohort effects, but instead a constant and linear change of incidence trends.

Similarly, the modelling analysis of ICC showed a constant and linear change of incidence rates and no significant period and cohort effects. In both sexes, the model with the best fit to the data was the age–drift model (data not shown).

### 3.2. Survival

From 2003 to 2017, as shown in the upper section of Table 4, men exhibited a constant and significant increase in total liver cancer survival that involved all indicators. For women too, a generalised improvement occurred, although the changes were of weaker significance.

This temporal pattern was strongly influenced by trends in survival from HCC (second section of Table 4). For men, 1-year, 2-year, 5-year, 10-year as well as 5|1-year and 5|2-year CNS from HCC all improved significantly. For women, increases were observed in 2- to 10-year NS, in 5|1-year CNS and, at a borderline level of significance, in 5|2-year CNS. In the last time period, 1-year and 5-year NS rates were above 65% and near 30%, respectively, in both sexes.

With respect to ICC (third section of Table 4), all survival probabilities were considerably lower in both sexes. In particular, the 5-year NS figures (men: 13.9%; women: 17.4%) were approximately half compared with those for HCC patients. However, an upward trend occurred in 2-year, 5-year and 10-year NS, which was of borderline significance for men and more pronounced for women.

The bottom section of Table 4 shows that, overall, survival estimates for patients with ‘other liver cancer types’ were nearly the same as those for patients with ICC. In particular, 5-year NS in the last study period was 16.6% for men and 17.5% for women. For men, there were significant increases in 1-year, 2-year, 5-year, 10-year NS and in 5|1-year and 5|2-year NS. For women, some kind of improvement was found in all indicators, although no variation reached the level of statistical significance.

**Figure 6:**
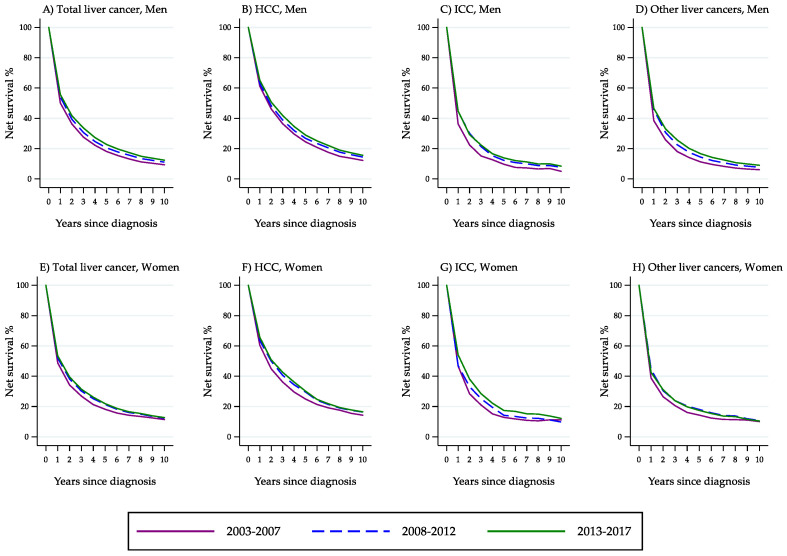
Percent net survival from total liver cancer, hepatocellular carcinoma, intrahepatic cholangiocarcinoma and ‘other liver cancer types’ by year since diagnosis and time period of diagnosis (Italy, 2003–2017).

Figure 6 shows the curves of NS probability from total liver cancer, HCC, ICC and ‘other liver cancer types’ by year since diagnosis and time period of diagnosis (men: panels A, B, C, D; women: panels E, F, G, H). The graphs illustrate in detail the changes in net survival probability by year since diagnosis.

## 4. Discussion

### 4.1. Main Findings

In brief, the above results indicate that: (i) between 2003 and 2017, the age-standardised incidence rate of HCC decreased constantly for both sexes, while ICC followed an opposite trend; (ii) the risk of HCC for men had two peaks, one in the cohorts of 1928 and 1933 and the other—less pronounced—in the cohort of 1958, with a slightly lower figure for the cohort of 1963; (iii) with respect to HCC, men exhibited an improvement in all survival indicators, and significant increases in 2- to 10-year NS and 5|1-year CNS were also observed among women; (iv) in the most recent study years, 5-year NS from HCC rose to about 30% for men as well as women; and (v) with respect to ICC, survival probabilities were lower but a general upward trend was observed.

### 4.2. Incidence

In Italy, there have been two time periods of intense HCV transmission [26]. In the central decades of the past century, before the introduction of disposable syringes and routine screening for HCV in blood transfusions and blood products, a peak was seen in the iatrogenic transmission of blood-borne viruses. This aetiologic route was especially frequent in southern Italy [26], a geographic area poorly represented in this study. The 1970s and 1980s, conversely, were characterised by the diffusion of intravenous drug use among people born after World War II. This aetiologic pattern was more common among men and in the north of the country. The coexistence of two transmission patterns was confirmed by the molecular characterisation of infecting HCVs [45].

Our findings are well consistent with this historical background. We can confirm that, after the peak occurring in the cohorts of 1928 and 1933, the risk of HCC decreased in subsequent cohorts of men due to the depletion of the pool of individuals iatrogenically infected with HCV. The incidence grew again until the first decade of this century for the cohort of 1958, as a consequence of the growing intravenous drug use in the 1970s and in the early 1980s [26]. The risk was only slightly lower in the cohort of 1963 and then decreased more sharply. As a consequence of this complex bimodal pattern, most of the generations included in this study exhibited a decreasing risk of HCC. This explains the decrease in the overall age-standardised incidence rate over the years, and it can be anticipated that this downtrend will not change substantially in the near future.

The observed downtrend in the incidence of HCC reflects not only a decreasing prevalence of exposure to viral infections (of different origin), but also improvements in the surveillance and treatment of both HBV- and HCV-infected patients. In the past, the standard of care consisted of prolonged treatment courses with interferon and ribavirin, which came with a high risk of failure and discontinuation because of frequent adverse effects. Progress has been made, however, and direct-acting antiviral agents (DAAs) have become the first-line treatment in spite of their high cost. DAAs achieve very high rates of sustained virological response with rare adverse events, even in advanced cirrhosis [46]. This is associated with a 70% reduced risk of HCC [5], although an increase persists because of the underlying chronic liver disease [46]. In the U.K. and Australia, the incidence of HCC in the DAAs’ era has plateaued [47,48]. In the United States, screening guidelines for persons born during 1945-1965 combined with these therapeutic advances are expected to accelerate the progress in reducing liver cancer mortality [18].

HBV vaccination is another cornerstone of public health policies to prevent viral hepatitis-related HCC. The World Health Organisation has set a 90% vaccination target to achieve HBV elimination globally by 2030. The HBV vaccine (the first cancer-preventing vaccine) is highly effective in preventing HBV transmission when the full three-or four-dose vaccination schedule is given at birth and in early infancy [49]. In Italy, HBV vaccination has been mandatory since 1991.

Finally, improvements in alcohol quality and a reduction in alcohol consumption have led to a steady decline in the incidence of, and mortality from, cirrhosis in Italy [50] and have conceivably had a beneficial effect on the risk of progression to liver cancer [26]. The national age-standardised liver cirrhosis mortality rate in 1980 was in the upper tertile on a global scale. By 2010, the rate ranked in the lowest tertile.

The present and previous Italian data are consistent with much international literature. HCC incidence rates are now plateauing or decreasing in many countries worldwide, at least in the male population [2,5,13,14,15,16,17]. In the United States, for example, a well-defined cohort-dependent decrease in HCC incidence started in the first decade of this century involving men and women aged 40–59 years [17].

Due to the multifaceted aetiology of HCC, current trends at the global level have multiple concurrent explanations, i.e., reductions in the risk of transfusion-transmitted HCV infection, increasing awareness of high-risk behaviours, improvements in HBV vaccination programmes, advances in the treatment of both HBV and HCV infections, and reduced exposure to aflatoxin. In Italy, the proportion of liver cancer that is due to HCV infection is as high as 60% versus a global average of 19% [28], and the prevalence of HCV increases to about 80% in injecting drug users [50,51]. This means that, in Italy, hepatitis C control plays a comparatively more important role in the prevention of HCC than elsewhere. In Australia, for example, both an increased migration from endemic HBV countries and a growing prevalence of metabolic syndrome, non-alcoholic fatty liver disease and type 2 diabetes were considered key risk factors for the uptrend of past decades [12]. Similarly, the leading cause of the increasing trend in liver cancer in the U.K. was HCV infection, but exposure to metabolic risk factors, aflatoxin B1 and tobacco grew more rapidly [5]. In these countries, emphasis has been placed on the fact that, although the risk of HCC from viral hepatitis is higher than that from obesity and alcohol, these two conditions have a much higher prevalence in the population [5]. Italy is also currently characterised by a rapidly increasing proportion of liver cancers that are due to causes other than the most common ones (HBV, HCV and excessive alcohol consumption) [28].

The results of this study are also consistent with many reports worldwide, including a previous report from Italy [52] showing that ICC is following an incidence trend in the opposite direction compared with HCC [6,19]. The dissociation of incidence trends, the difference in the men-to-women IRR and the evidence that the risk of ICC depends on a wider spectrum of factors (including those not identified yet) are objective arguments in favour of the view that the web of causation differs between the two diseases [4,5,6,7,8,9,10,11]. Equally important, the aetiological peculiarities of ICC seem to be associated with different levels of clinical aggressiveness, since tumour stage distribution and prognosis of ICC are worse [20], which is also the case for Italy. At diagnosis, about 60% of patients with ICC have locally advanced disease or metastatic disease and, as a consequence, a high proportion of them fail to receive any cancer-specific therapy [11].

### 4.3. Survival

Expectedly, we found a considerably higher survival probability for patients with HCC as compared with patients with ICC. For the former, we also found a significant increase in all survival indicators among men and in most indicators among women. Between-sex differences in survival from HCC were modest.

The finding that men and women exhibited comparable improvements in both early and mid-term survival from HCC indicates that the trend towards an earlier detection, albeit important, was coupled with improvements in curative as well as supportive treatments. Since many patients with advanced-stage HCC die in the first two years after diagnosis, improvements in 5|1-year and particularly in 5|2-year NS reflect the impact of variation in adjuvant treatments of patients with no evidence for distant metastasis at diagnosis [53]. In the past two decades, advances have especially been made in systemic chemotherapy, with the introduction of sorafenib, multiple embolisation, ablation surgery and liver transplantation, which may provide a cure not only for the cancer, but also for the underlying liver disease [5]. Another key innovation is that clinical decisions are increasingly taken on a multidisciplinary basis—a work model that has been demonstrated to improve survival in several areas of cancer care. A closely linked hypothesis has been raised in the Netherlands, where the increasing survival trend has been interpreted as resulting from the implementation of national guidelines recommending to centralise the diagnosis and treatment of HCC [54]. There is robust evidence that receiving treatment at a high-volume centre is positively associated with survival from non-metastatic HCC with local or regional spread, possibly in relation to multidisciplinary evaluation, greater subspecialty expertise and access to more treatment modalities [55]. In Italy, during the past decade, stringent hospital and volume criteria have been developed for identifying the referral centres entitled to perform liver surgery [56].

Increases in survival from HCC need to be considered with a view to the lead time bias, a potential correlate of patient surveillance and earlier diagnosis of the disease [5]. In particular, according to estimates, the lead time bias would account for part of the short-term (one-year) benefit provided by surveillance for HCC, whereas the benefit becomes true in the long term [57]. This confirms the clinical utility of an earlier detection of HCC compared with clinical diagnosis by symptoms.

For patients with ICC, the survival rates were poorer. Among men, however, we observed improvements of borderline statistical significance in 1-year, 2-year, 5-year and 10-year NS. Among women, there were comparable changes but at a higher level of significance. Studies from other countries have reported improvements in survival from ICC varying from moderate [24,25] to non-significant [19]. Substantial improvements have been obtained only among patients undergoing surgical treatment [23].

The poorer prognosis of ICC depends primarily on two concomitant adverse conditions: the disease remains asymptomatic for long time [5] and is not readily susceptible to surveillance programmes aimed at preclinical detection or prompt clinical detection. This suggests that the survival disadvantage affecting patients with ICC compared with HCC may be explained, at least in part, with a more advanced stage at diagnosis. A recent international clinical study has shown that a significant proportion of patients fail to receive any cancer-specific therapy [11].

The partly different trends in survival from HCC and ICC could also be interpreted in the light of aetiology differences. HBV and HCV infections account for most HCC cases and, in Italy, the attributable risk is particularly high [28]. As a consequence, the proportion of HCC cases diagnosed with surveillance for HBV and HCV and cirrhosis has selectively increased, which has led to an earlier and more curable tumour stage and an increased curability of the underlying chronic liver disease [5]. This trend might account for the overall fair level of survival from liver cancer in Italy and is among the reasons why it would be preferable to monitor the epidemiology of the two main types of liver cancer as distinct entities.

The different prognosis of HCC and ICC may have important implications. First, the opposite incidence trends of the two types, with an absolute and relative increase in ICC rates, may theoretically erode, in the long run, the ongoing (albeit slow) increase in survival from liver cancer as a whole [22]. Second, as stated above, HCV infection is more closely associated with HCC than ICC. Consequently, the higher proportion of liver cancers that are attributable to HCV infection in Italy [28] might be the reason for the uncommonly high proportion of HCCs we observed among men, which was 89% of cases with known morphology. In turn, this might offer a potential explanation for the comparatively high survival of Italian patients in international studies [22]. Given the substantial prevalence of liver cancer cases with unknown morphological code, however, these hypotheses need to be considered with some caution.

As a final remark, we emphasise that our survival data are consistent with those of the international study by Allemani et al., who reported an overall 5-year NS of Italian patients with liver cancer (i.e., all types included and both sexes combined) of 20.0% in 2005–2009 and 20.3% in 2010–2014 [22]. We evaluated HCC, ICC and ‘other liver cancer types’ as independent entities. For comparison purposes, however, we estimated the overall 5-year NS from total liver cancer in the present study and found a very similar figure, that is, 20.5% (data not shown). As the study by Allemani et al. included a 3.5-fold larger number of Italian registries, this similarity of results supports the representativeness of our population and the validity of our findings.

### 4.4. ‘Other Liver Cancer Types’

Cases lacking histologic verification constituted the greater part of liver cancers classified as ‘other liver cancer types’ in this study. Compared with HCC and ICC, this group showed an apparently contradictory pattern, with an incidence trend similar to that of HCC and survival probabilities similar to those of patients with ICC. The relative rarity of ICC in Italy, however, makes it very likely that most of the cases in this group had an undetected hepatocellular morphology. This hypothesis is supported by the decreasing incidence trend, while the poor survival could be explained assuming that the lack of histological verification was related to advanced disease stage and tumour unresectability.

### 4.5. Methodological Considerations

There are some methodological issues that need to be addressed. First, the quality of data for liver cancer in population-based cancer registries is known to be highly variable [53]. The high, but varying, proportion of DCO registrations makes survival estimates for liver cancer patients less reliable and less comparable than for most patients with other solid cancer types [22]. It must be considered that incomplete trace-back work to ascertain the date of diagnosis of DCO cases may bias survival upwards, since DCO cases are often diagnosed shortly before death. In the entire period of this study, the DCOs share was 2.0%. The same proportion was maintained in 2008-2012, when the average figure of the other European cancer registries included in *Cancer Incidence in Five Continents* Volume XI was 9.0% [30]. This makes it unlikely that the high survival rates seen in Italy were upwardly biased by DCO cases.

Second, some of the between-registry differences in liver cancer registration are due to coding variability and misclassification errors, among which the inaccurate assignments of cancer cases to primaries versus metastases (and vice versa) are the most serious ones. International registration rules usually recommend not using specific morphologic codes (for example, ‘hepatocarcinoma’) [32] in the absence of a microscopic confirmation [58]. In fact, several patients cannot be biopsied for adverse clinical conditions. Other biases may arise from the topographical exact attribution of some cancers of the bile ducts (intrahepatic/extrahepatic). The paradigm of this bias is represented by the Klatskin tumour, an extrahepatic bile duct tumour that ICD-O-3 classification allows to classify both as intra- or extrahepatic [4]. Today, imaging has reached a good level of accuracy and in high-income countries it has become the main diagnostic technique [59]. This allows contaminations between liver primaries and metastases to be excluded. For the same reason, cases without microscopic confirmation include HCC and ICC in about the same proportion as biopsied lesions [4]. Finally, in our data, Klatskin tumours represent only 3.5% of all cancers of the bile ducts. This figure is well within the range observed elsewhere [60,61] and has little chance to warp incidence and survival data.

Third, the lack of information on tumour stage should be regarded as a severe limitation of this study. In Italy, tumour stage is not among the standard registration variables, except for some specialised registries [62]. The use of multiple prognostic indicators, however, allowed us to separate the impact of early deaths—due to late-stage, rapidly fatal cancers—from the impact of more delayed fatalities caused by clinically less advanced cancers with occult metastases at diagnosis.

### 4.6. Policy Implications

Despite the favourable direction of incidence and survival trends, the burden of liver cancer, and particularly of HCC, is still high. Given the etiologic role of exposure to HCV infection [28], it appears that the avoidance of high-risk behaviours, the treatment of HCV infections and the surveillance of HCV-infected persons remain priorities, especially in Italy.

Until a substantial decrease in incidence has been obtained, however, it is necessary that the efforts to improve patient survival through secondary prevention and treatment are intensified [53]. The finding that all HCC survival indicators used in this study improved, especially among men, is encouraging in that it indicates that improvements have been made in both tumour stage at diagnosis and in the effectiveness of adjuvant and supportive treatments.

In terms of public health strategies, there is large consensus among liver cancer stakeholders that the highest priority should be given to the monitoring of at-risk populations, the creation of centres of excellence, multidisciplinary management, national guidelines, public awareness and access to treatments. With respect to the biomedical research agenda, in the mid-term, it will become increasingly necessary to pay due attention to the subset of liver cancer cases attributable to uncommon and unclear causes [28], as well as to ICC, the incidence of which follows an upward and opposite trend to that of HCC.

## 5. Conclusions

In Italy, during the last two decades, progress has been made in both sexes in reducing the incidence of HCC and improving patient survival. For the much less frequent ICC, incidence is still on the rise and survival is lower but increasing. 

## Figures and Tables

**Table 1:** Cancer registries participating in the study, population covered, period of diagnosis, liver cancer cases contributed, crude and age-standardised rate and data quality indexes (Italy, 2003–2017).

Cancer Registry	Population *	Period of Diagnosis	Incident Cases	Crude Rate ^†^	Age-Standardised Rate ^†,‡^	Microscopic Verification (%)	DCO (%)
South Tyrol	527,750	2003–2017	1241	16.4	17.8	40%	2.2%
Ferrara	349,692	2003–2017	1266	23.8	18.2	32%	1.0%
Firenze-Prato	1,266,471	2003–2016 ^§^	2277	14.5	12.4	39%	2.0%
Friuli Venezia Giulia	1,212,809	2003–2017	4498	24.7	20.9	50%	1.0%
Genova	846,211	2003–2016	2534	20.9	15.9	35%	3.4%
Modena	702,949	2003–2017	1923	18.7	17.3	46%	1.0%
Parma	448,207	2003–2017	2009	31.0	27.0	43%	0.5%
Piacenza	287,246	2006–2017	1035	30.1	24.4	34%	2.3%
Puglia	2,778,877	2006–2017	6486	19.2	19.1	30%	3.7%
Reggio Emilia	533,392	2003–2017	1251	16.2	15.8	39%	1.0%
Romagna	1,125,415	2003–2017	2174	13.4	11.9	41%	1.6%
Trento	538,604	2003–2017	1646	21.3	21.0	46%	1.5%
Veneto	2,125,066	2003–2017	7234	22.9	21.4	41%	2.0%
All cancer registries	12,742,689		35,574	20.2	18.3	40%	2.0%

DCO, death certificate only. * In 2017, except for the registries of Firenze-Prato and Genova (2016). ^†^ Per 100,000 persons. ^‡^ Age-standardised to the 2013 European standard population. ^§^ The data collection by the registry of Firenze-Prato was not done in 2012.

**Table 2:** Number and age-standardised incidence rate of total liver cancer, hepatocellular carcinoma, intrahepatic cholangiocarcinoma and ‘other liver cancer types’, by period of diagnosis, and percent distribution by histologic type (Italy, 2003–2017).

Sex and Type	2003–2017				2003–2007			2008–2012			2013–2017		
	No.	%	Rate *	(95% CI)	No.	Rate *	(95% CI)	No.	Rate *	(95% CI)	No.	Rate *	(95% CI)
Men													
Total	24,692	100.0%	29.4	(29.0; 29.8)	7519	31.7	(31.0; 32.4)	8790	30.1	(29.5; 30.8)	8383	27.0	(26.4; 27.6)
HCC	12,343	50.0%	14.6	(14.3; 14.8)	3757	15.6	(15.1; 16.1)	4339	14.7	(14.3; 15.2)	4247	13.6	(13.2; 14.1)
ICC	1531	6.2%	1.8	(1.7; 1.9)	351	1.4	(1.3; 1.6)	513	1.7	(1.6; 1.9)	667	2.2	(2.0; 2.3)
Other	10,818	43.8%	13.0	(12.8; 13.3)	3411	14.7	(14.2; 15.2)	3938	13.7	(13.2; 14.1)	3469	11.2	(10.9; 11.6)
Women													
Total	10,882	100.0%	9.4	(9.2; 9.6)	3496	10.4	(10.0; 10.7)	3853	9.5	(9.2; 9.8)	3533	8.4	(8.1; 8.7)
HCC	3881	35.7%	3.5	(3.4; 3.6)	1309	4.0	(3.8; 4.2)	1356	3.5	(3.3; 3.7)	1216	3.0	(2.8; 3.2)
ICC	1151	10.6%	1.1	(1.0; 1.2)	269	0.9	(0.8; 1.0)	411	1.1	(1.0; 1.2)	471	1.2	(1.1; 1.4)
Other	5850	53.8%	4.8	(4.7; 5.0)	1918	5.5	(5.3; 5.8)	2086	4.9	(4.7; 5.2)	1846	4.2	(4.0; 4.4)

CI, confidence interval; HCC, hepatocellular carcinoma; ICC, intrahepatic cholangiocarcinoma. * Per 100,000 persons, age-standardised to the 2013 European standard population.

**Table 3:** Comparison of age–period–cohort models of liver cancer incidence, by histologic type and sex (Italy, 2003–2017).

Type	Sex	Terms in the Model	Degrees of Freedom	Deviance	AIC	Models to Compare	Deviance Difference	Degrees of Freedom Difference	*p*-Value
HCC	Men	Age (A)	16	67.9	11.363				
		Age–drift (Ad)	15	43.5	10.429	Ad versus A	24.4	1	<0.001
		Age–period (AP)	14	42.0	10.448	AP versus Ad	25.9	2	0.215
		**Age–cohort (AC)**	**7**	**7.5**	**9.594**	**AC versus Ad**	**60.5**	**9**	**<0.001**
		Age–period–cohort (APC)	6	6.9	9.655	APC versus AP	35.1	8	<0.001
						APC versus AC	0.5	1	0.462
	Women	Age (A)	16	69.5	9.990				
		**Age–drift (Ad)**	**15**	**19.1**	**7.973**	**Ad versus A**	**50.4**	**1**	**<0.001**
		Age–period (AP)	14	18.7	8.040	AP versus Ad	50.8	2	0.532
		Age–cohort (AC)	7	3.3	7.982	AC versus Ad	66.2	9	0.046
		Age–period–cohort (APC)	6	2.6	8.036	APC versus AP	16.1	8	0.041
						APC versus AC	0.7	1	0.404
ICC	Men	Age (A)	16	48.7	8.418				
		**Age–drift (Ad)**	**15**	**12.4**	**6.989**	**Ad versus A**	**36.3**	**1**	**<0.001**
		Age–period (AP)	14	12.3	7.070	AP versus Ad	36.4	2	0.811
		Age–cohort (AC)	7	8.0	7.472	AC versus Ad	40.7	9	0.820
		Age–period–cohort (APC)	6	7.7	7.544	APC versus AP	4.6	8	0.799
						APC versus AC	0.3	1	0.600
	Women	Age (A)	16	31.0	7.374				
		**Age–drift (Ad)**	**15**	**10.5**	**6.605**	**Ad versus A**	**20.5**	**1**	**<0.001**
		Age–period (AP)	14	8.8	6.616	AP versus Ad	22.2	2	0.186
		Age–cohort (AC)	7	3.1	6.963	AC versus Ad	27.9	9	0.492
		Age–period–cohort (APC)	6	2.2	7.008	APC versus AP	6.6	8	0.581
						APC versus AC	0.9	1	0.337

A, age; AC, age–cohort; Ad, age–drift; AIC, Akaike information criterion; AP, age–period; APC, age–period–cohort. For each disease and sex group, the model with the best fit to the data is indicated in bold.

**Table 4:** Percent 1-year, 2-year, 5-year and 10-year net survival and 5|1-year and 5|2-year conditional net survival from total liver cancer, hepatocellular carcinoma, intrahepatic cholangiocarcinoma and ‘other liver cancer types’ by sex and period of diagnosis (Italy, 2003–2017).

Type	Sex	Time since Diagnosis		Net Survival, % (95% CI)		
			2003–2007	2008–2012	2013–2017	*p*-Value *
Total liver cancer	Men	1 year	50.1 (49.0; 51.3)	54.1 (52.9; 55.2)	55.7 (54.5; 56.9)	<0.001
		2 years	36.2 (35.1; 37.4)	39.4 (38.2; 40.5)	41.7 (40.5; 42.9)	<0.001
		5 years	18.1 (17.1; 19.1)	20.5 (19.5; 21.6)	22.7 (21.6; 23.9)	<0.001
		10 years	9.3 (8.5; 10.2)	11.1 (10.2; 12.0)	12.4 (11.4; 13.4)	<0.001
		5|1 year	35.3 (33.5; 37.0)	37.3 (35.6; 39.1)	39.8 (38.1; 41.5)	<0.001
		5|2 years	48.4 (46.1; 50.6)	51.3 (49.2; 53.4)	53.7 (51.4; 55.9)	<0.001
	Women	1 year	48.8 (46.6; 51.0)	52.0 (49.9; 54.0)	53.4 (51.3; 55.5)	0.212
		2 years	34.2 (32.0; 36.4)	38.2 (36.1; 40.2)	39.5 (37.3; 41.6)	0.023
		5 years	18.2 (16.3; 20.2)	21.4 (19.5; 23.3)	21.9 (20.0; 23.8)	0.007
		10 years	11.4 (9.7; 13.3)	12.4 (10.8; 14.1)	12.7 (11.1; 14.5)	0.014
		5|1 year	35.9 (32.7; 39.0)	39.4 (36.6; 42.3)	39.4 (36.6; 42.3)	<0.001
		5|2 years	50.6 (46.7; 54.3)	53.4 (50.1; 56.7)	53.0 (49.2; 56.6)	0.032
Hepatocellular carcinoma	Men	1 year	61.2 (59.6; 62.8)	63.0 (61.3; 64.5)	65.2 (63.5; 66.7)	0.001
		2 years	46.2 (44.5; 47.9)	48.3 (46.6; 50.0)	50.7 (49.0; 52.4)	<0.001
		5 years	24.5 (22.9; 26.0)	26.8 (25.2; 28.4)	28.9 (27.3; 30.6)	<0.001
		10 years	12.2 (11.0; 13.6)	14.4 (13.0; 15.9)	15.6 (14.1; 17.2)	<0.001
		5|1 year	39.3 (37.1; 41.6)	42.3 (40.0; 44.5)	43.9 (41.7; 46.1)	0.002
		5|2 years	51.8 (49.0; 54.5)	55.0 (52.4; 57.5)	56.4 (53.5; 59.1)	0.009
	Women	1 year	60.5 (56.9; 63.9)	63.6 (60.5; 66.5)	65.7 (62.4; 68.8)	0.177
		2 years	44.9 (41.2; 48.4)	49.9 (46.6; 53.1)	50.8 (47.3; 54.2)	0.035
		5 years	24.9 (21.6; 28.4)	29.6 (26.4; 32.8)	30.1 (26.9; 33.5)	0.007
		10 years	14.3 (11.3; 17.6)	16.4 (13.5; 19.4)	16.5 (13.7; 19.6)	0.023
		5|1 year	41.0 (37.0; 44.9)	44.5 (40.4; 48.5)	44.9 (40.8; 48.9)	0.011
		5|2 years	53.4 (48.6; 58.0)	56.2 (51.6; 60.6)	57.5 (52.5; 62.2)	0.070
Intrahepatic cholangiocarcinoma	Men	1 year	36.3 (31.6; 40.9)	44.4 (40.1; 48.6)	44.8 (40.9; 48.6)	0.090
		2 years	22.4 (18.2; 26.9)	30.0 (25.9; 34.2)	29.2 (25.6; 33.0)	0.059
		5 years	9.7 (6.7; 13.3)	12.3 (9.2; 15.8)	13.9 (10.8; 17.3)	0.057
		10 years	5.0 (2.9; 8.1)	7.7 (5.1; 11.0)	8.5 (5.6; 12.0)	0.052
		5|1 year	27.3 (19.1; 36.2)	27.1 (20.5; 34.2)	30.7 (24.2; 37.5)	0.367
		5|2 years	43.7 (31.2; 55.6)	40.2 (30.8; 49.5)	45.9 (34.8; 56.3)	0.682
	Women	1 year	46.7 (41.0; 52.2)	47.0 (42.2; 51.7)	54.1 (49.5; 58.5)	0.159
		2 years	28.4 (23.1; 33.8)	33.1 (28.5; 37.8)	38.1 (33.5; 42.7)	0.032
		5 years	12.8 (9.1; 17.2)	14.2 (10.7; 18.1)	17.4 (13.5; 21.7)	0.037
		10 years	11.2 (6.8; 16.7)	9.8 (6.5; 13.9)	12.2 (8.3; 16.8)	0.040
		5|1 year	28.5 (20.2; 37.3)	30.3 (23.2; 37.8)	32.3 (25.3; 39.4)	0.116
		5|2 years	47.1 (34.1; 58.9)	43.2 (33.5; 52.4)	46.1 (35.9; 55.7)	0.645
Other liver cancer types	Men	1 year	38.5 (36.8; 40.2)	45.7 (43.9; 47.4)	46.6 (44.7; 48.4)	0.001
		2 years	25.7 (24.1; 27.4)	30.6 (28.9; 32.3)	33.0 (31.1; 34.9)	<0.001
		5 years	11.2 (9.9; 12.6)	14.4 (13.0; 15.8)	16.6 (14.9; 18.4)	<0.001
		10 years	6.1 (5.0; 7.3)	7.7 (6.5; 9.0)	9.0 (7.6; 10.6)	<0.001
		5|1 year	28.2 (25.3; 31.2)	30.7 (28.0; 33.5)	34.2 (31.2; 37.1)	0.002
		5|2 years	41.7 (37.7; 45.6)	46.0 (42.3; 49.7)	49.3 (45.3; 53.2)	0.008
	Women	1 year	38.8 (35.6; 42.0)	44.5 (41.2; 47.8)	42.5 (39.1; 46.0)	0.568
		2 years	26.5 (23.3; 29.7)	30.4 (27.3; 33.7)	31.1 (27.8; 34.5)	0.282
		5 years	14.3 (11.6; 17.3)	18.1 (15.2; 21.1)	17.5 (14.6; 20.5)	0.253
		10 years	10.1 (7.5; 13.0)	10.8 (8.3; 13.7)	10.4 (8.0; 13.3)	0.284
		5|1 year	34.3 (28.9; 39.7)	39.0 (34.0; 43.9)	39.2 (34.2; 44.2)	0.160
		5|2 years	50.0 (43.3; 56.3)	56.4 (50.6; 61.8)	51.5 (44.7; 57.9)	0.785

CI, confidence interval. * From the Wald test for trend in the exponential of the period of diagnosis coefficient. The period of diagnosis was entered as a continuous regressor in a Poisson regression model for net survival.

## Data Availability

The anonymised dataset used in this study is available on request from the corresponding author.

## Supplementary Materials

The following supporting information can be downloaded at https://www.mdpi.com/article/10.3390/cancers14246162/s1; Figure S1: Geographic distribution of the 13 cancer registries participating in the study on trends in liver cancer incidence and survival by histologic type (Italy, 2003–2017).

## Appendix A

**Table A1:** Incidence rate ratio of hepatocellular carcinoma in the male population and 95% confidence intervals, as estimated with an age–cohort model, by birth cohort (Italy, 2003–2017).

**Birth Cohort**	**Incidence Rate Ratio**	**(95% CI)**
1923	0.92	(0.85; 1.00)
1928	1.00	(reference)
1933	1.00	(0.95; 1.05)
1938	0.90	(0.83; 0.97)
1943	0.83	(0.76; 0.91)
1948	0.72	(0.65; 0.80)
1953	0.66	(0.58; 0.75)
1958	0.76	(0.65; 0.88)
1963	0.74	(0.62; 0.88)
1968	0.61	(0.48; 0.78)

CI, confidence interval.
