# Supplementary material for: Trends in Liver Cancer Incidence and Survival in Italy by Histologic Type, 2003–2017
Source: Cancers (Basel). 2022 Dec 14;14(24):6162. doi: 10.3390/cancers14246162 (PMC9777051; doi:10.3390/cancers14246162)
Supplement: Supplementary file 1 [file cancers-14-06162-s001.zip › cancers-1984440-supplementary_revised_R2.pdf]

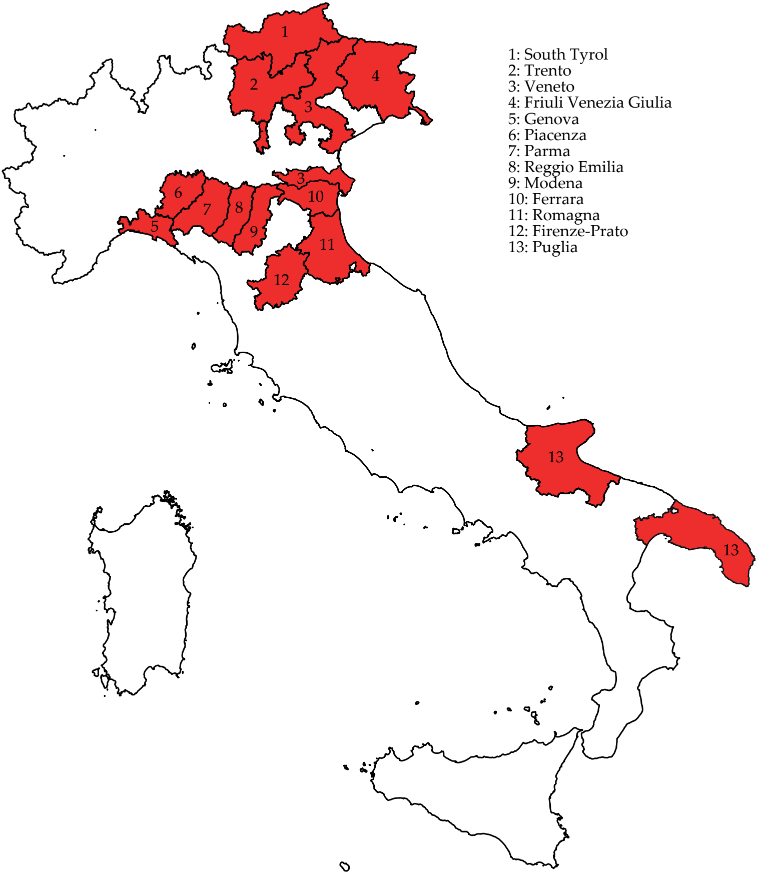

Figure S1. Geographic distribution of the 13 cancer registries participating in the study on trends in liver cancer incidence and survival by histologic type (Italy, 2003-2017). In the administrative Regions of Veneto and Puglia, the area of coverage by the local cancer registry was incomplete and included two separate, nonadjacent zones.
